# The Impact of Comorbidity Burden on The Association between Vascular Access Type and Clinical Outcomes among Elderly Patients Undergoing Hemodialysis

**DOI:** 10.1038/s41598-019-54191-1

**Published:** 2019-12-03

**Authors:** Jong Hyun Jhee, Seun Deuk Hwang, Joon Ho Song, Seoung Woo Lee

**Affiliations:** 1 Division of Nephrology, Department of Internal Medicine, Gangnam Severance Hospital, Yonsei University College of Medicine, Seoul, Korea; 2 Division of Nephrology and Hypertension, Department of Internal Medicine, Inha University, College of Medicine, Incheon, Korea

**Keywords:** Haemodialysis, Kidney diseases

## Abstract

The optimal vascular access type for elderly hemodialysis patients is controversial. We evaluated the impact of comorbidity burden on the association between vascular access type and mortality risk among 23,100 hemodialysis patients aged ≥65 years from the Korean Society of Nephrology End-Stage Renal Disease registry data. Subjects were stratified into tertiles according to the simplified Charlson comorbidity index (sCCI), and the survival and hospitalization rates were compared with respect to vascular access type: arteriovenous fistula (AVF), arteriovenous graft (AVG), and central venous catheter (CVC). Among all tertiles of sCCI, CVC use showed highest risk of mortality than AVF use. In the lowest to middle tertile, no difference was observed in survival rates between the use of AVF and AVG. However, in the highest tertile, AVG use showed higher risk of mortality than AVF use. When subjects were classified according to a combination of sCCI tertile and access type (AVF vs. AVG), patients with the highest CCI with AVG showed 1.75-folded increased risk of mortality than those with the lowest sCCI with AVF. Hospitalization rates due to access malfunction were highest in patients with CVC in all sCCI tertiles. In the highest tertile, patients with AVG showed increased rates of hospitalization compared to those with AVF due to access malfunction. However, hospitalization rates due to access infection were highest in patients with AVG in all tertiles. The use of AVF may be of benefit and switching to AVF should be considered in elderly hemodialysis patients with a high burden of comorbidity.

## Introduction

The prevalence of elderly patients with end-stage renal disease (ESRD) is increasing worldwide^1^. In Korea, the primary treatment for geriatric ESRD is hemodialysis (HD, 81.3% of elderly patients)^2^. The average age of dialysis patients was 61.2 ± 14.5 years in 2016, which has steadily increased from 55.2 years in 2005^3^. The percentage of elderly dialysis patients in Korea (over 65 years old) was 43.9% in 2016, representing a significant increase from 28% in 2005. Accordingly, the associated range of comorbidities and geriatric syndromes such as frailty is also increasing, and the higher burden of comorbidity makes elderly patients more prone to adverse outcomes after dialysis initiation^4–6^. Although nephrologists take great effort to improve their prognosis, there are still no clear guidelines established for adequate dialysis prescription in elderly dialysis patients.

The selection of the most optimal type of vascular access for elderly patients undergoing HD is controversial, and several underlying comorbidities make this selection difficult. In the majority of elderly patients, HD is initiated with a central venous catheter (CVC) with a subsequent switch to the placement of a permanent vascular access device, either an arteriovenous fistula (AVF) or an arteriovenous graft (AVG)^1^. Previous studies suggest that placement of an AVF is preferred over placement of an AVG^7,8^. These suggestions are based on the observation that AVFs result in better long-term survival and require fewer interventions to maintain their patency as compared with AVGs. However, the advantages of AVFs over AVGs may be less apparent in elderly patients undergoing HD. Maturation of AVF is much slower in elderly patients compared with younger patients, and the high burden of comorbidity in elderly patients may contribute to a high rate of AVF non-maturation^9,10^. In addition, elderly patients have shorter life expectancies, which means that they may not live long enough to see the benefits of prolonged AVF survival^11^.

Considering the range of comorbidities is thus crucial when choosing and maintaining the proper vascular access type in elderly patients undergoing HD. This study aimed to evaluate the impact of comorbidity burden on the association between vascular access type and mortality risk among elderly patients undergoing HD.

## Results

### Baseline characteristics

Baseline characteristics of the study subjects are presented in Table 1. The mean age was 73.7 ± 6.0 years, and 56.1% of patients were men. The mean of dialysis vintage was 3.8 ± 3.3 years, and the most common cause of ESRD among all study subjects was diabetic nephropathy (51.1%). We classified study subjects according to the type of vascular access: AVF (n = 14,847), AVG (n = 3,906), and CVC (n = 4,347). Patients using AVF or AVG tended to be younger, were more likely to be male, and had higher body mass index (BMI) and normalized protein catabolic rate (nPCR) compared with those using CVC. The mean dialysis vintage was shorter in patients with CVC than in patients with AVF or AVG. There were no differences in levels of single-pool Kt/V and predialysis systolic or diastolic blood pressures (SBP or DBP) among groups. Patients with AVF or AVG showed better functional status than those with CVC. The mean of the simplified CCI (sCCI) was 5.8 ± 1.1 among all patients. The sCCI was lowest in patients with AVF and highest in those with CVC (5.7 ± 1.1, 5.9 ± 1.1, and 6.0 ± 1.2 in AVF, AVG, and CVC, respectively, *P* < 0.001) However, patients using AVF or AVG had higher prevalence of diabetes, hypertension, and coronary artery disease but lower prevalence rates of cerebrovascular disease than those with CVC. In laboratory tests, patients with AVF or AVG showed higher levels of hemoglobin and serum albumin compared to those with CVC.

**Table 1:** Baseline characteristics according to type of vascular access.

	Total (n = 23,100)	Type of vascular access			*P*
		AVF (n = 14,847)	AVG (n = 3,906)	CVC (n = 4,347)	
**Demographic data**					
Age, years	73.7 ± 6.0	73.0 ± 5.7	74.3 ± 5.9	75.9 ± 6.6	<0.001
Gender, male	12,948 (56.1)	8,672 (58.4)	2,037 (52.2)	2,239 (51.5)	<0.001
BMI, kg/m^2^	22.2 ± 4.3	22.3 ± 3.8	21.9 ± 3.2	21.8 ± 6.7	<0.001
Dialysis vintage, year	3.8 ± 3.3	4.2 ± 3.4	3.7 ± 3.2	2.5 ± 2.6	<0.001
nPCR, g/kg/day	0.4 ± 0.9	0.4 ± 0.9	0.3 ± 0.6	0.3 ± 0.6	<0.001
Single-pool Kt/V	1.49 [1.28–1.78]	1.49 [1.28–1.77]	1.53 [1.32–1.79]	1.49 [1.26–1.82]	0.07
SBP, mmHg	138.9 ± 24.3	138.8 ± 24.9	139.8 ± 24.6	138.2 ± 21.2	0.08
DBP, mmHg	73.4 ± 16.3	13.6 ± 14.6	72.6 ± 23.9	73.7 ± 12.3	0.03
Functional status					<0.001
Inability to ambulate	1,508 (11.1)	603 (7.0)	268 (11.1)	637 (24.2)	
Need of assistance with daily activities	4,394 (32.3)	2,441 (28.5)	873 (36.3)	1,080 (41.0)	
Independently living	7,696 (56.2)	5,514 (64.4)	1,266 (52.6)	916 (34.8)	
Etiology of ESRD					<0.001
Diabetic nephropathy	11,804 (51.1)	7,696 (51.8)	2,047 (52.4)	2,061 (47.4)	
Hypertension	5,436 (23.5)	3,482 (23.5)	954 (24.4)	1,000 (23.0)	
Glomerulonephritis	1,327 (5.7)	920 (6.2)	166 (4.2)	241 (5.5)	
Polycystic kidney disease	275 (1.2)	198 (1.3)	44 (1.1)	33 (0.8)	
Other	1,329 (5.8)	741 (5.0)	204 (5.2)	384 (8.8)	
Unknown	2,929 (12.7)	1,810 (12.2)	491 (12.6)	628 (14.4)	
sCCI	5.8 ± 1.1	5.7 ± 1.1	5.9 ± 1.1	6.0 ± 1.2	<0.001
**Comorbidities**					
Hypertension	11,804 (51.1)	7,696 (51.8)	2,047 (52.4)	2,061 (47.4)	<0.001
Diabet®es	9,027 (39.1)	5,984 (40.3)	1,604 (41.1)	1,439 (33.1)	<0.001
Coronary artery disease	3,136 (13.6)	2,077 (14.0)	538 (13.8)	521 (12.0)	0.002
Congestive heart failure	1,563 (6.8)	968 (6.5)	279 (7.1)	316 (7.3)	0.05
Cerebrovascular disease	1,428 (6.2)	867 (5.8)	254 (6.5)	307 (7.1)	0.002
Malignancy	656 (2.8)	413 (2.8)	99 (2.5_	144 (3.3)	0.14
**Laboratory findings**					
Hemoglobin, g/dL	10.1 ± 3.0	10.2 ± 3.2	10.2 ± 3.2	9.8 0 ± 1.4	<0.001
Albumin, g/dL	3.8 ± 0.6	3.9 ± 0.6	3.8 ± 0.6	3.5 ± 0.7	<0.001
Total cholesterol, mg/dL	142.7 ± 39.5	142.5 ± 38.7	142.7 ± 38.9	143.5 ± 42.7	0.55
HbA1c, %	6.6 ± 7.2	6.7 ± 8.5	6.4 ± 2.5	6.3 ± 4.7	0.19
Calcium, mg/dL	8.7 ± 0.8	8.7 ± 0.9	8.7 ± 0.8	8.5 ± 0.9	<0.001
Phosphorous, mg/dL	4.3 ± 1.4	4.4 ± 1.4	4.2 ± 1.4	4.0 ± 1.5	<0.001
intact-PTH, pg/mL	160.0 ± 148.7	162.6 ± 152.8	150.1 ± 132.4	159.9 ± 148.2	0.001

Data were presented as mean ± standard deviation, median [interquartile range] or number (%).

*Abbreviation:* AVF, arteriovenous fistula; AVG, arteriovenous graft; CVC, central venous catheter; BMI, body mass index; ESRD, end-stage renal disease; sCCI, simplified Charlson comorbidity index; PTH, parathyroid hormone.

### Current state of vascular access use according to the comorbidity burden

**Figure 1:**
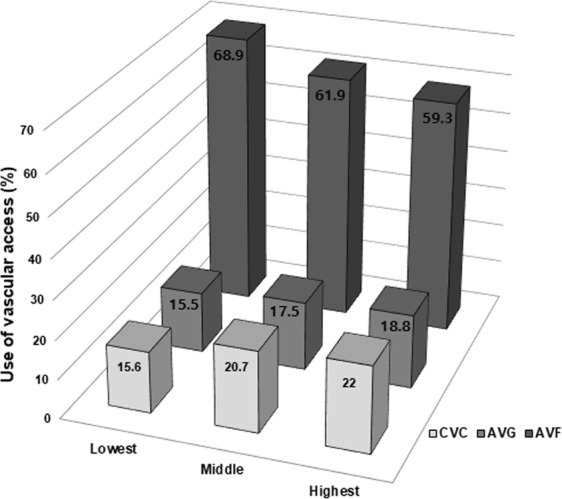
Percentage use of vascular access according to tertiles of sCCI. X axis represents tertiles of sCCI, *P* for trend <0.001. *Abbreviation:* CVC, central venous catheter; AVG, arteriovenous graft; AVF, arteriovenous fistula; sCCI, simplified Charlson comorbidity index.

Next, we compared the current state of vascular access use according to sCCI. In all cases, the prevalence of AVF use was the highest (68.9%, 61.9%, and 59.3% in the lowest, middle, and highest sCCI groups). However, in higher sCCI groups, the use of AVF was lower, and the use of AVG and CVC was significantly higher (Fig. 1).

### Vascular access type and all-cause mortality

**Figure 2:**
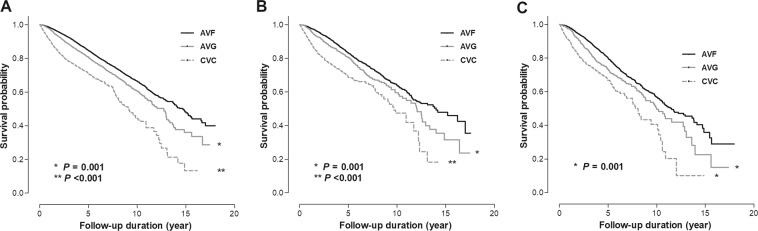
Kaplan-Meier curve for the risk of all-cause mortality according to type of vascular access st different levels of sCCI. *Abbreviation:* CVC, central venous catheter; AVG, arteriovenous graft; AVF, arteriovenous fistula; sCCI, simplified Charlson comorbidity index.

We assessed the association between vascular access type and all-cause mortality according to sCCI groups. During a median follow-up of 3.8 [2.1–6.1] years, 4,405 (19.1%) deaths occurred. First, we plotted Kaplan-Meier curves for the risk of all-cause mortality according to the type of vascular access at different levels of sCCI (Fig. 2A–C). In all cases, the use of AVF showed the best survival rates, and the use of CVC showed the worst rates. In multivariable Cox analysis after adjustment for age, sex, dialysis vintage, BMI, predialysis SBP, etiology of ESRD, functional status, history of hypertension, diabetes, or cardiovascular diseases (CVDs), hemoglobin, serum albumin, and HbA1c, the use of CVC still showed the highest risk of all-cause mortality in all sCCI groups compared with the use of AVF. However, the use of AVG showed a greater risk of all-cause mortality than AVF only in patients in the highest sCCI group (hazard ratio [HR], 1.33; 95% confidence interval [CI], 1.02–1.75, *P* = 0.04) (Table 2).

**Table 2:** The association between type of vascular access and all-cause mortality risk at different level of sCCI.

	Model 1		Model 2		Model 3	
	HR (95% CI)	*P*	HR (95% CI)	*P*	HR (95% CI)	*P*
**Lowest tertile of sCCI (4–5)**						
AVF (n = 6,748)	1.00 (reference)		1.00 (reference)		1.00 (reference)	
AVG (n = 1,515)	1.29 (1.12–1.48)	<0.001	1.24 (1.08–1.42)	0.002	1.04 (0.67–1.63)	0.86
CVC (n = 1,528)	4.05 (3.54–4.63)	<0.001	2.45 (2.14–2.81)	<0.001	2.81 (1.90–4.16)	<0.001
**Middle tertile of sCCI (6)**						
AVF (n = 5,041)	1.00 (reference)		1.00 (reference)		1.00 (reference)	
AVG (n = 1,423)	1.29 (1.12–1.47)	<0.001	1.27 (1.11–1.24)	0.001	1.20 (0.88–1.65)	0.25
CVC (n = 1,685)	3.38 (3.00–3.82)	<0.001	2.30 (2.03–2.60)	<0.001	2.54 (1.83–3.53)	<0.001
**Highest tertile of sCCI (≥7)**						
AVF (n = 3,058)	1.00 (reference)		1.00 (reference)		1.00 (reference)	
AVG (n = 968)	1.39 (1.21–1.60)	<0.001	1.27 (1.10–1.26)	0.001	1.33 (1.00–1.75)	0.04
CVC (n = 1,134)	3.22 (2.81–3.69)	<0.001	2.01 (1.75–2.30)	<0.001	1.43 (1.08–1.90)	0.01

Model 1: Unadjusted.

Model 2: Adjusted for age and sex.

Model 3: Model 2 + dialysis vintage, BMI, predialysis SBP, etiology of ESRD, functional status, history of hypertension, diabetes, or CVDs, hemoglobin, serum albumin, and HbA1c.

*Abbreviation:* sCCI, simplified Charlson comorbidity index; HR, hazard ratio; CI, confidence interval; AVF, arteriovenous fistula; AVG, arteriovenous graft; CVC, central venous catheter; BMI, body mass index; SBP, systolic blood pressure; ESRD, end-stage renal disease; CVD, cardiovascular disease.

### Comparison of the use of AVF vs. AVG according to the comorbidity burden

**Figure 3:**
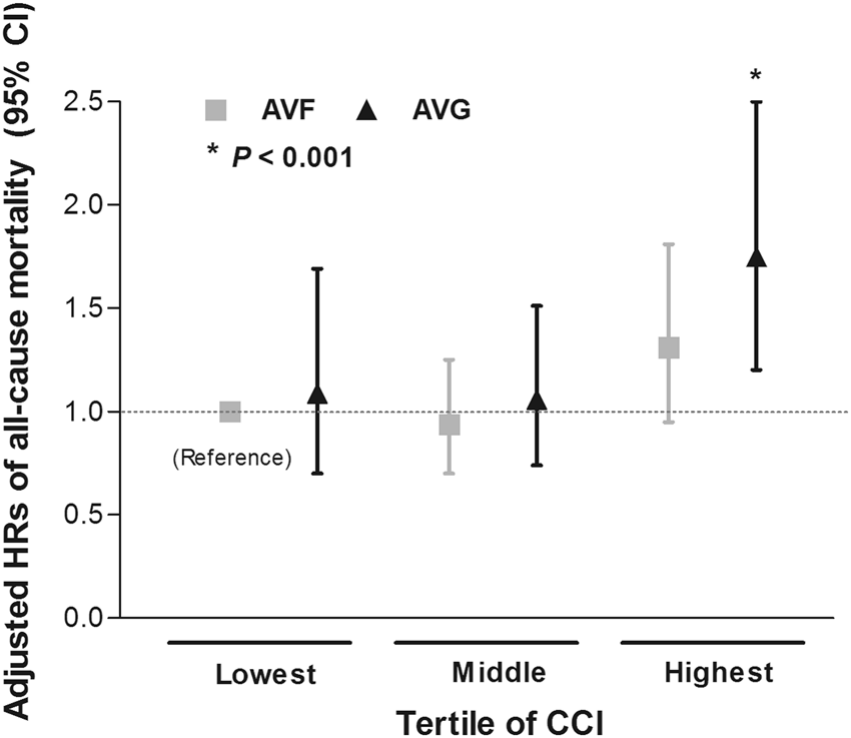
The adjusted risk for all-cause mortality according to combination group of sCCI tertile and the type of vascular access (AVF vs. AVG). ^*^Model was adjusted for age, sex, dialysis vintage, body mass index, predialysis systolic blood pressure, etiology of end-stage renal disease, functional status, history of hypertension, diabetes, or cardiovascular diseases, hemoglobin, serum albumin, and HbA1c. *Abbreviation:* AVF, arteriovenous fistula; AVG, arteriovenous graft; sCCI, simplified Charlson comorbidity index; CI, confidence interval.

Next, we further compared the risk of mortality between the use of AVF and AVG according to sCCI tertiles. We formed six groups based on the combination of AVF or AVG and sCCI tertiles. The incidence of all-cause mortality was higher in higher sCCI groups. Moreover, in all equivalent sCCI groups, the use of AVG showed higher mortality rates than the use of AVF (Supplementary Table 1). Multivariable Cox analysis was performed after adjustment for confounding factors, and the results indicated that patients using AVG in the highest sCCI tertile was associated with 75% increased risk of all-cause mortality compared with those using AVF in the lowest sCCI (HR, 1.75; 95% CI, 1.20–2.50, *P* = 0.004) (Fig. 3).

### Vascular access type and hospitalization rate according to comorbidity burden

To evaluate the association between the rate of hospitalization and vascular access type according to the sCCI, we performed logistic regression analysis (Table 3). The number of hospitalizations due to all causes was highest in patients using CVC, followed by AVG and AVF. We separately analyzed the risk of hospitalization based on the cause, that is, either access malfunction or access infection. The total events of hospitalization due to access malfunction occurred in 1,061 cases (4.6%) and that of hospitalization due to access infection occurred in 180 cases (0.8%). The risk of hospitalization due to access malfunction was highest in patients using CVC compared with those using AVF in all sCCI tertiles. However, while no association was observed between patients with AVG in the lowest to middle sCCI groups and those with AVF, patients with AVG in the highest sCCI group showed a significantly increased risk of hospitalization due to access malfunction (odds ratio [OR], 1.74; 95% CI 1.04–2.92; *P* = 0.03). In contrast, the risk of hospitalization due to access infection was not associated with the use of CVC in any sCCI tertiles, whereas the use of AVG was significantly associated with increased risk in all CCI tertiles compared with the use of AVF (HR, 4.41; 95% 1.35–14.45 in lowest sCCI, HR, 5.00; 95% CI, 1.57–15.90 in middle sCCI, and HR, 3.90; 95% CI, 1.47–10.32 in highest sCCI tertile).

**Table 3:** The association between type of vascular access and hospitalization at different level of sCCI.

	Number of hospitalizations^†^	Access malfunction			Access infection		
	Mean ± SD	Case (%)^†^	OR (95% CI)^*^	*P*	Case (%)^†^	OR (95% CI)^*^	*P*
Lowest tertile of sCCI (4–5)		375 (3.8)			57 (0.6)		
AVF (n = 6,748)	0.6 ± 2.4	184 (2.7)	1.00 (reference)		29 (0.4)	1.00 (reference)	
AVG (n = 1,515)	0.7 ± 1.2	79 (5.2)	1.65 (0.89–3.03)	0.11	15 (1.0)	4.41 (1.35–14.45)	0.01
CVC (n = 1,528)	1.0 ± 1.3	112 (7.3)	2.83 (1.62–4.94)	<0.001	13 (0.9)	1.53 (0.35–6.65)	0.57
Middle tertile of sCCI (6)		395 (4.8)			57 (0.7)		
AVF (n = 5,041)	0.8 ± 1.7	181 (3.6)	1.00 (reference)		18 (0.40	1.00 (reference)	
AVG (n = 1,423)	1.0 ± 2.8	74 (5.2)	1.27 (0.80–2.04)	0.31	24 (1.7)	5.00 (1.57–15.90)	0.01
CVC (n = 1,685)	1.2 ± 2.3	140 (8.3)	2.22 (1.41–3.48)	0.001	15 (0.9)	2.74 (0.58–12.85)	0.20
Highest tertile of sCCI (≥7)		291 (5.6)			66 (1.3)		
AVF (n = 3,058)	1.1 ± 1.4	120 (3.9)	1.00 (reference)		19 (0.6)	1.00 (reference)	
AVG (n = 968)	1.2 ± 1.3	67 (6.9)	1.74 (1.04–2.92)	0.03	29 (3.0)	3.90 (1.47–10.32)	0.01
CVC (n = 1,134)	1.5 ± 1.4	104 (9.2)	3.76 (2.31–6.11)	<0.001	18 (1.6)	1.27 (0.31–5.22)	0.74

^†^*P* for trend < 0.001.

*Models were adjusted for age, sex, dialysis vintage, BMI, predialysis SBP, etiology of ESRD, functional status, history of hypertension, diabetes, or CVDs, hemoglobin, serum albumin, and HbA1c.

*Abbreviation:* sCCI, simplified Charlson comorbidity index; OR, odds ratio; CI, confidence interval; AVF, arteriovenous fistula; AVG, arteriovenous graft; CVC, central venous catheter; BMI, body mass index; SBP, systolic blood pressure; ESRD, end-stage renal disease; CVD, cardiovascular disease.

## Discussion

This study compared the risk of all-cause mortality and hospitalization rates due to catheter malfunction or infection based on maintenance of vascular access type at different levels of comorbidity burden in elderly patients undergoing HD. As sCCI increased, the use of AVF was lower, while use of AVG and CVC was higher. In all sCCI tertiles, the use of CVC was associated with the highest risk of all-cause mortality compared to the use of AVF. The use of AVG vs. AVF showed no differences in risk of mortality in the lowest to middle sCCI tertiles. However, in the highest sCCI tertile, the use of AVG showed a higher risk of all-cause mortality than the use of AVF. Furthermore, hospitalization rates due to access malfunction were higher with the use of CVC in all sCCI tertiles, whereas hospitalization rates due to access infection were higher with use of AVG compared with use of AVF.

Currently, the international clinical practice guidelines recommend a “fistula first” approach based on the best long-term outcomes, lowest mortality, and lowest health care costs of AVF compared with AVG and CVC^12–15^. Several observational studies with elderly patients also demonstrated superior survival rates in those using AVF compared with AVG and CVC^16,17^. However, another study reported that there is no significant survival benefit in the different vascular access types^18^. Recently, Lee *et al*.^19^ suggested tradeoffs in vascular access selection in elderly patients initiating HD. They suggested that the use of AVF, compared with the use of AVG, is less likely to be successful after initiation, more likely to require interventions to make it functional, and associated with longer CVC dependence. In contrast, AVF requires fewer interventions to maintain patency after successful access creation. Hence, the optimal type of vascular access for elderly patients undergoing HD is still controversial, and several factors make selection of the best type of access difficult. In elderly patients undergoing HD, the geriatric barriers to dialysis that make selection of vascular access type difficult should be taken into consideration. These include the impact of age, functional status, vessel suitability for access creation, maturation, complications, expected access survival, the competing risk of death for dialysis initiation, and the burden of comorbidity^20^.

Multiple comorbidities have a great impact on the vasculature, especially on the vessel wall^21^. The presence of vascular calcification especially reduces the patency of a vascular access and leads to poor clinical outcomes^22^. Lee *et al*.^23^ reported that pre-existing arterial pathologic changes such as atherosclerosis affect not only the patency of vascular access but also increase the risk of cardiovascular mortality. These poor conditions of the vessel wall have an even greater effect when patients are concomitant with diabetes or coronary artery disease^22,24^. The degree of comorbidities should thus be taken into account when selecting the most optimal type of vascular access, especially in elderly patients who have a high burden of comorbidity. No studies to date have examined the effect of disease burden on the association between vascular access type and clinical outcomes. In the current study, we showed that the use of CVC resulted in the greatest increase in the risk of all-cause mortality regardless of the degree of comorbidity. However, the use of AVG resulted in inferior survival rates compared with the use of AVF only in patients with the highest sCCI. When the use of AVF and AVG was directly compared with sCCI tertiles, the risk of mortality was highest in those with a high sCCI using AVG. Several explanations can be deduced as follows. First, as previous studies suggested, AVGs require more interventions to maintain patency after access creation as compared with AVFs^19^. As shown in our study, hospitalization rates due to access malfunction were significantly increased with the use of AVG compared to the use of AVF only in the highest sCCI group. Some studies suggested that the use of AVG might be more beneficial in elderly patients, because these patients might not survive long enough to benefit from AVFs^25–27^. They argued that the advantages of AVFs over AVGs are not seen immediately. Although AVGs require more interventions to maintain patency, AVFs require more interventions to mature; therefore, overall patency might not differ substantially between the two types of permanent access^28,29^. This remains controversial, and questions still surround the selection of the most optimal type of access for elderly patients. Based on the results of our study, we suggest that elderly patients with high comorbidities should maintain AVF use or switch to AVF use if they are using AVG, as elderly patients tend to require frequent vascular interventions. Second, the infection rate is much higher with the use of AVG compared to the use of AVF^25,30^. It is evident from the previous study results that the key risk factor for vascular access–associated infection is the type of access itself^31^. Most studies showed that the risk of vascular access infection is highest with CVC use and decreases with use of AVG and AVF^32–34^. In addition, increasing age or multiple comorbid conditions are also significant risk factors for vascular access infection^35^. In our study, hospitalization rates due to access infection were highest with the use of AVG compared with the use of AVF as well as CVC across all sCCI groups. It is unclear why hospitalization rates due to access infection were higher with use of AVG than with use of CVC, a finding which is contrary to previous study results. However, Ravani *et al*. recently reported that the risk for infectious complication were highest immediately after placement of CVC, but this risk declined and become similar among all types of vascular access over time^36^. In our study, the vascular access types were investigated after several years of their creation in majority of subjects and the total event rates of access infection were significantly low (180 cases, 0.8%). Thus, the cautious interpretation is needed that the risk of infections complication can show different results from that of initial creation of vascular access. Furthermore, we assumed that the patients using CVC had more comorbidities and were at the greatest risk of death, and that this risk outweighed the risk of access infection. Nevertheless, the use of AVG showed a higher risk of infection than the use of AVF. We thus suggest that elderly patients who are prone to infection should use AVF or switch to AVF from AVG. Finally, patients using AVG, especially those with a high burden of comorbidity, were at an increased risk of mortality themselves and could not have afforded to create the AVF. In our study, the trend of using AVF was decreased, whereas using AVG or CVC was increased as the sCCI increases. This implies that high burden of comorbidities hinder to create AVF with native vessels and, conversely, the use of AVG represents being encountered with worse clinical outcomes.

This study has several limitations. First, due to the retrospective study design, a substantial number of patients with missing vascular access or comorbidity data were excluded from the analysis. Furthermore, selection bias cannot be excluded that clinician likely to have selected a certain vascular access type based on patient characteristics. For instance, clinicians tend to use AVF in patients with less comorbidities and with expectation to survive long. In this respect, the results could be driven by those characteristics as opposed to solely by vascular access types. Thus, we made multiple adjustment models and classified subjects according to comorbidity burden to minimize this bias. Nevertheless, the cautious generalization of our findings to the HD population is needed because there might be still unmeasured confounders. Second, the information about vascular access type was investigated at the enrollment of Korean Society of Nephrology End-Stage Renal Disease (KSN ESRD) registry, not at the initiation of dialysis. Hence, conversion of vascular access to another access type could not be investigated. Most studies focused on access type at the initiation of dialysis or immediately after initiation. However, our study has clinical implications, since we evaluated the effect of maintaining vascular access on outcomes. Specifically, if elderly patients with high morbidity maintain vascular access with AVG or CVC, immediate conversion to AVF should be suggested. Third, the comorbidity index was assessed with limited value compared to the original CCI, because there was a lack of information on past history such as hemiplegia, specific cancer types, connective tissue disease, and acquired immune deficiency syndrome (AIDS) in the KSN ESRD registry. However, overall prevalence rates of these diseases were comparably low, which may have had little effect on the main outcomes. Finally, the rate of mortality in our cohort could have been underestimated, because death reports are collected voluntarily, and the causes of death were extracted from patient records.

In conclusion, in elderly patients undergoing HD with a high burden of comorbidity, the use of AVF may be of greater benefit than AVG with respect to survival rates and lower hospitalization rates due to access-associated infections. Moreover, the use of CVC is associated with increased risk of mortality and higher hospitalization rates due to access malfunction. Thus, in elderly patients using CVC during HD, the change to use of AVF should be considered, and in elderly patients with multiple morbidities using AVG, the change to AVF should be considered for better survival and clinical prognosis.

## Methods

### Study subjects

This study used data from the KSN ESRD registry, a nationwide database of medical records of patients with ESRD from 2001 to 2018. The ESRD registry committee of the KSN launched the official ESRD patient registry in 1985. The registry data were collected through an internet program that was opened in 2001 and revised in 2013 (http://www.ksn.or.kr)^37^. A total of 111,964 patients undergoing HD were initially screened, and 23,100 elderly patients aged ≥65 years were eventually analyzed, excluding cases with insufficient data and those which provided no data on vascular access (Supplementary Fig. 1). The use of vascular access at the time of study enrollment was investigated, and subjects were classified into three groups according to access type: AVF, AVG, and CVC. All participants were enrolled in the study voluntarily, and informed consent was obtained in all cases. This study was carried out in accordance with the Declaration of Helsinki and was approved by the Institutional Review Board of Inha University Hospital (INHAUH201812015).

### Anthropometric and laboratory data

Demographic and clinical data were collected at the time of study enrollment. Age, sex, BMI, dialysis vintage, nPCR, HD adequacy (single-pool Kt/V), SBP and DBP, functional status, cause of ESRD, and comorbidities including diabetes, hypertension, coronary artery disease, congestive heart failure, cerebrovascular disease, and malignancy were recorded. All laboratory data were based on initial values entered in the KSN registry.

### Assessment of the simplified Charlson comorbidity index

We assessed comorbidity burden based on CCI. The comorbidities that make up the CCI are myocardial infarction, congestive heart failure, peripheral vascular disease, cerebrovascular disease, dementia, chronic pulmonary disease, connective tissue disease, peptic ulcer disease, liver disease, diabetes with or without end-organ damage, hemiplegia, moderate to severe CKD, tumors including solid tumors, leukemia, or lymphoma, and AIDS^38^. However, owing to lack of information in the study database, we modified original CCI and developed sCCI as follow; the diagnosis of connective tissue disease, hemiplegia, and AIDS was not included in the KSN ESRD database, we excluded these items. Furthermore, specific tumor type was not investigated, we assigned a score of 2 for any tumor type. Since all patients in our dataset had ESRD, we assigned a score of 2 for any subjects and the lowest possible sCCI score was 2. Information on comorbidities were obtained by clinicians from the KSN ESRD registry through patient histories and reviews of electronic health records based on International Classification of Diseases, 10^th^ Revision codes at the time of study enrollment^39,40^.

### Study outcomes

The primary outcome measure was all-cause mortality, and the secondary outcome was hospitalization rate due to catheter related malfunction or infection. Of note, there was a possibility of over-estimation of patient survival due to the voluntary nature of registration in the ESRD registry, with submission of death reports could have easily been missed.

### Statistical analysis

All statistical analyses were performed with IBM SPSS software for Windows version 23.0 (IBM Corporation, Armonk, NY, USA) and R software 3.3.1 (http://www.R-project.org). Continuous variables were expressed as mean ± standard deviation and categorical variables as absolute numbers with percentages. All data were tested for normality before statistical analysis. The Kolmogorov–Smirnov test was performed to determine the normality of the distribution of parameters. Comparisons between groups were performed using analysis of variance or Student’s t-test for continuous variables with normal distributions and the chi-square test or Fisher’s exact test for categorical variables. Data that did not show a normal distribution were presented as medians with interquartile ranges and were compared using the Mann–Whitney U-test or the Kruskal–Wallis test. Cumulative survival rates were estimated with the Kaplan–Meier analysis and a log-rank test. Survival time was defined as the time interval between baseline and either the occurrence of outcomes or the last follow-up. Patients who died or were lost to follow-up were censored at the date of the last examination. Cox proportional hazards models were constructed to determine the independent predictive value of vascular access types in mortality. Logistic regression analysis was performed to evaluate the association between vascular access types and the risk of hospitalization. Variables that showed statistical significance in univariable analyses or were considered to have clinical significance were included in multivariable models. All results were expressed as HRs or ORs and 95% CI. For all analyses, a *P* value < 0.05 was considered statistically significant.

## Supplementary information

Supplementary Material

## Data Availability

Data is available as supplementary information.

## References

[1] Saran, R. *et al*. US Renal Data System 2014 Annual Data Report: Epidemiology of Kidney Disease in the United States. *American journal of kidney diseases: the official journal of the National Kidney Foundation* **66**, Svii, S1–305 (2015). 10.1053/j.ajkd.2015.05.001 PMC6643986 26111994

[2] Lee S (2014). An assessment of survival among Korean elderly patients initiating dialysis: a national population-based study. PloS one.

[3] Jin DC (2018). Current characteristics of dialysis therapy in Korea: 2016 registry data focusing on diabetic patients. Kidney research and clinical practice.

[4] O’Hare AM (2007). Age affects outcomes in chronic kidney disease. Journal of the American Society of Nephrology: JASN.

[5] Kurella M, Covinsky KE, Collins AJ, Chertow GM (2007). Octogenarians and nonagenarians starting dialysis in the United States. Annals of internal medicine.

[6] Sejoong K (2019). Time-varying effects of body mass index on mortality among hemodialysis patients: Results from a nationwide Korean registry. Kidney Res Clin Pract.

[7] Clinical practice guidelines for vascular access. *American journal of kidney diseases: the official journal of the National Kidney Foundation* **48** Suppl 1, S176–247 (2006). 10.1053/j.ajkd.2006.04.029 16813989

[8] Allon M, Robbin ML (2002). Increasing arteriovenous fistulas in hemodialysis patients: problems and solutions. Kidney international.

[9] Richardson AI (2009). Should fistulas really be first in the elderly patient?. The journal of vascular access.

[10] Lok CE (2006). Risk equation determining unsuccessful cannulation events and failure to maturation in arteriovenous fistulas (REDUCE FTM I). Journal of the American Society of Nephrology: JASN.

[11] O’Hare AM (2007). When to refer patients with chronic kidney disease for vascular access surgery: should age be a consideration?. Kidney international.

[12] Tordoir J (2007). EBPG on Vascular Access. Nephrology, dialysis, transplantation: official publication of the European Dialysis and Transplant Association - European Renal Association.

[13] Jindal K (2006). Hemodialysis clinical practice guidelines for the Canadian Society of Nephrology. Journal of the American Society of Nephrology: JASN.

[14] Clinical practice guidelines for vascular access. *American journal of kidney diseases: the official journal of the National Kidney Foundation* 48 Suppl 1, S248–273 (2006). 10.1053/j.ajkd.2006.04.040 16813991

[15] Polkinghorne KR (2013). KHA-CARI Guideline: vascular access - central venous catheters, arteriovenous fistulae and arteriovenous grafts. Nephrology (Carlton, Vic.).

[16] Ocak G (2011). Haemodialysis catheters increase mortality as compared to arteriovenous accesses especially in elderly patients. Nephrology, dialysis, transplantation: official publication of the European Dialysis and Transplant Association - European Renal Association.

[17] Lee T, Thamer M, Zhang Q, Zhang Y, Allon M (2017). Vascular Access Type and Clinical Outcomes among Elderly Patients on Hemodialysis. Clinical journal of the American Society of Nephrology: CJASN.

[18] Jakes AD (2016). Arterio-Venous Fistula: Is it Critical for Prolonged Survival in the over 80’s Starting Haemodialysis?. PloS one.

[19] Lee T, Qian J, Thamer M, Allon M (2018). Tradeoffs in Vascular Access Selection in Elderly Patients Initiating Hemodialysis With a Catheter. American journal of kidney diseases: the official journal of the National Kidney Foundation.

[20] Viecelli AK, Lok CE (2019). Hemodialysis vascular access in the elderly-getting it right. Kidney international.

[21] Letachowicz K (2016). Vascular access should be tailored to the patient. Seminars in vascular surgery.

[22] Choi SJ (2015). Pre-existing Arterial Micro-Calcification Predicts Primary Unassisted Arteriovenous Fistula Failure in Incident Hemodialysis Patients. Seminars in dialysis.

[23] Lee JY, Kim YO (2017). Pre-existing arterial pathologic changes affecting arteriovenous fistula patency and cardiovascular mortality in hemodialysis patients. The Korean journal of internal medicine.

[24] Chitalia N (2015). Neointimal hyperplasia and calcification in medium sized arteries in adult patients with chronic kidney disease. Seminars in dialysis.

[25] Tamura MK, Tan JC, O’Hare AM (2012). Optimizing renal replacement therapy in older adults: a framework for making individualized decisions. Kidney international.

[26] Moist LM (2012). Optimal hemodialysis vascular access in the elderly patient. Seminars in dialysis.

[27] O’Hare AM (2013). Vascular access for hemodialysis in older adults: a “patient first” approach. Journal of the American Society of Nephrology: JASN.

[28] Lok CE (2013). Cumulative patency of contemporary fistulas versus grafts (2000–2010). Clinical journal of the American Society of Nephrology: CJASN.

[29] Lok CE (2005). Arteriovenous fistula outcomes in the era of the elderly dialysis population. Kidney international.

[30] Praga M (2013). Type of vascular access and survival among very elderly hemodialysis patients. Nephron. Clinical practice.

[31] Lafrance JP, Rahme E, Lelorier J, Iqbal S (2008). Vascular access-related infections: definitions, incidence rates, and risk factors. American journal of kidney diseases: the official journal of the National Kidney Foundation.

[32] Stevenson KB (2002). Epidemiology of hemodialysis vascular access infections from longitudinal infection surveillance data: predicting the impact of NKF-DOQI clinical practice guidelines for vascular access. American journal of kidney diseases: the official journal of the National Kidney Foundation.

[33] Tokars JI, Miller ER, Stein G (2002). New national surveillance system for hemodialysis-associated infections: initial results. American journal of infection control.

[34] Hoen B, Paul-Dauphin A, Hestin D, Kessler M (1998). EPIBACDIAL: a multicenter prospective study of risk factors for bacteremia in chronic hemodialysis patients. Journal of the American Society of Nephrology: JASN.

[35] Jean G (2002). Risk factor analysis for long-term tunneled dialysis catheter-related bacteremias. Nephron.

[36] Ravani P (2013). Temporal risk profile for infectious and noninfectious complications of hemodialysis access. Journal of the American Society of Nephrology: JASN.

[37] Jin DC (2015). Dialysis registries in the world: Korean Dialysis Registry. Kidney international supplements.

[38] Charlson ME, Pompei P, Ales KL, MacKenzie CR (1987). A new method of classifying prognostic comorbidity in longitudinal studies: development and validation. Journal of chronic diseases.

[39] Charlson M, Szatrowski TP, Peterson J, Gold J (1994). Validation of a combined comorbidity index. Journal of clinical epidemiology.

[40] Quan H (2005). Coding algorithms for defining comorbidities in ICD-9-CM and ICD-10 administrative data. Medical care.

