# Supplementary material for: The Impact of Comorbidity Burden on The Association between Vascular Access Type and Clinical Outcomes among Elderly Patients Undergoing Hemodialysis
Source: Sci Rep. 2019 Dec 3;9:18156. doi: 10.1038/s41598-019-54191-1 (PMC6890785; doi:10.1038/s41598-019-54191-1)
Supplement: Supplementary file 1 — Supplementary Material [file 41598_2019_54191_MOESM1_ESM.docx]

**ONLINE SUPPORTING MATERIAL**

**The Impact of Comorbidity Burden on The Association between Vascular Access Type and Clinical Outcomes among Elderly Patients Undergoing Hemodialysis**

Jong Hyun Jhee M.D.^1^, Seun Deuk Hwang M.D.^2^, Joon Ho Song M.D., Ph.D. ^2^, and Seoung Woo Lee M.D., Ph.D. ^2^

^1^Division of Nephrology, Department of Internal Medicine, Gangnam Severance Hospital, Yonsei University College of Medicine, Seoul, Korea

^2^Division of Nephrology and Hypertension, Department of Internal Medicine, Inha University

College of Medicine, Incheon, Korea

**Running head:** Comorbidity burden and vascular access type on clinical outcomes

**Corresponding Author:**

Seoung Woo Lee, M.D., Ph.D.

Division of Nephrology and Hypertension, Department of Internal Medicine, Inha University

College of Medicine, Inha University Hospital, 27 Inhang-ro, Jung-gu, Incheon, 23322, Republic

of Korea

Phone: 82-32-890-2228

Fax: 82-32-882-6578

E-mail: swleemd@inha.ac.kr


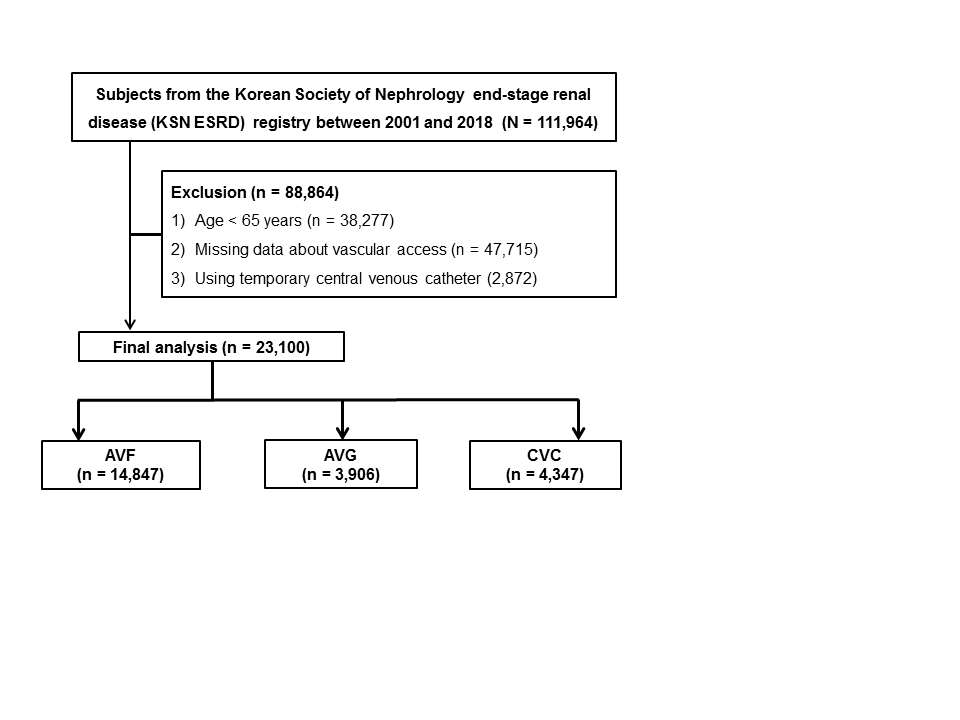


**Supplementary Figure 1.** Study subjects

***Abbreviation:*** AVF, arteriovenous fistula; AVG, arteriovenous graft; CVC, central venous catheter

**Supplementary Table 1.** Comparison of the incidence of all-cause mortality among six groups^*^

|  | CCI tertile | | |
| --- | --- | --- | --- |
|  | Lowest | Middle | Highest |
| Vascular access type, n (%) |  |  |  |
| AVF | 981 (14.5) | 880 (17.5) | 752 (24.6) |
| AVG | 256 (16.9) | 278 (19.5) | 255 (26.3) |
| ^*^ *P* for trend < 0.001  *Abbreviation:* CCI, charlson comorbidity index; AVF, arteriovenous fistula; AVG, arteriovenous graft; CVC, central venous catheter | | | |
